# Cytokine Profiling for the Prediction of Lethality and High-Dose Exposure in a Murine Partial Body Irradiation Model

**DOI:** 10.3390/ijms27073213

**Published:** 2026-04-01

**Authors:** Wanchang Cui, Lisa Hull, Asher Rothstein, Li Wang, Bin Lin, Min Zhai, Alia Weaver, Mang Xiao

**Affiliations:** 1 Armed Forces Radiobiology Research Institute, Uniformed Services University of the Health Sciences, Bethesda, MD 20814, USA; 2 Department of Pharmacology and Molecular Therapeutics, Uniformed Services University of the Health Sciences, Bethesda, MD 20814, USA; 3 Henry M Jackson Foundation for the Advancement of Military Medicine, Inc., Bethesda, MD 20817, USA; 4 Department of Pathology, Uniformed Services University of the Health Sciences, Bethesda, MD 20814, USA

**Keywords:** radiation biodosimetry, partial-body irradiation, serum cytokines, exposure prediction, survival prediction, dose discrimination

## Abstract

Accurate radiation biodosimetry is urgently needed for medical management after large-scale radiation exposure. Partial-body irradiation with 5% bone marrow sparing (PBI/BM5) provides a realistic radiation model. The current study specifically focused on the high-dose lethality window (12–16 Gy), where survival transitioned from 100% to 0%, representing a clinically distinct and underserved scenario requiring dedicated biodosimetry tools. We defined the survival profile of male C57BL/6 mice after PBI/BM5 and found that doses of 13.5–14.0 Gy were nonlethal within 12 days, whereas 15.0–15.5 Gy caused 100% mortality within 12 days, with a calculated LD_50/12_ of 14.68 Gy. A separate cohort of 14.0 Gy showed 100% survival up to 90 days post-radiation. To develop serum cytokine-based biodosimetry in high-dose radiation exposure, mice were exposed to 12.0–16.0 Gy PBI/BM5, and serum was collected on days 1, 3, and 7. A multiplex cytokine assay was used to quantify 70 total cytokines/chemokines. After the exclusion of 4 targets outside detection limits, 66 markers were utilized for downstream analysis. PCA, clustering and heatmaps, and LASSO classification revealed that cytokine signatures can classify radiation groups/status/doses. A 4-cytokine panel (IL-7, GDF-15, IL-16 and FLT3L) could distinguish naïve vs. irradiated mice on all study days. A 24-cytokine signature panel distinguished radiation survivors vs. non-survivors, and another 34-cytokine panel separated radiation doses (12–16 Gy); the prediction was better on day 7 compared to earlier time points. This exploratory study was specifically designed to define the systemic inflammatory response in a high-dose window where survival transitions from 100% to 0% (the ‘lethality threshold’) in a clinically relevant partial-body irradiation model. These findings show that serum cytokines have strong potential for high-dose triage, survival prediction, and dose discrimination within the near-lethal exposure range in a clinically relevant PBI/BM5 model. Extension to lower dose ranges is an important direction for future work.

## 1. Introduction

Rapid and accurate radiation biodosimetry is essential for effective medical triage and clinical management following accidental or intentional radiation exposure [1,2]. In mass casualty scenarios, biodosimetry tools must reliably identify exposed individuals, estimate dose, and predict clinical outcome in order to guide timely medical interventions [3]. Although significant progress has been made in the development of radiation biodosimetry approaches, there remains a critical gap between experimental models and real-world exposure conditions.

Current biodosimetry strategies include cytogenetic assays [4], gene expression profiling [5], and protein-based biomarkers [6]. Cytogenetic methods such as dicentric chromosome analysis remain the gold standard for dose estimation but are labor-intensive, time-consuming, and poorly suited for rapid large-scale screening [4]. Gene expression-based approaches have shown promise but often require specialized processing, are sensitive to RNA stability, and may be influenced by technical and biological variability [5]. In contrast, protein biomarkers measured in blood offer practical advantages for field-deployable and clinical biodosimetry, particularly when rapid turnaround and scalability are required [7].

A major limitation of many biodosimetry studies is their reliance on ex vivo radiation of human blood or on total-body irradiation (TBI) animal models. Human blood ex vivo radiation cannot capture the whole-body response, such as inflammatory cytokine changes. While TBI provides experimental simplicity and uniform exposure, it does not accurately represent most real-world radiation scenarios, where exposure is typically heterogeneous and partial-body in nature. Partial-body irradiation (PBI) models that spare a fraction of bone marrow better reflect accidental, occupational, or radiological incident exposures and more closely recapitulate clinical radiation injury [8,9]. PBI with 5% bone marrow sparing (PBI/BM5) allows for survival at high radiation doses while preserving hematopoietic recovery, thereby enabling the study of dose-dependent injury, survival, and delayed effects under realistic exposure conditions [10].

Within this context, circulating cytokines represent an attractive class of biodosimetric biomarkers. Cytokines are rapidly released in response to radiation-induced tissue damage, inflammation, and immune activation, and their concentrations can be readily quantified in serum or plasma using multiplexed platforms [11,12]. Importantly, cytokines reflect integrated systemic responses to radiation injury across multiple organs, rather than isolated cellular damage, making them well-suited for assessing both exposure and biological effect [7]. Several cytokines, including FLT3 ligand (FLT3L), interleukins, and acute-phase proteins, have been shown to respond dynamically to radiation in a dose- and time-dependent manner, supporting their utility for radiation exposure assessment and outcome prediction [6,13]. Our own data have shown serum cytokines such as IL-18 and GDF-15 have great potential to reflect radiation damage [14,15].

Despite these advantages, most cytokine-based biodosimetry studies have been conducted using TBI models, limiting their translational relevance [16]. The performance of cytokine signatures in clinically relevant PBI models, particularly across a range of nonlethal and lethal doses, remains insufficiently characterized. Moreover, the temporal dynamics of cytokine responses and their ability to distinguish exposed versus non-exposed individuals, as well as survivors versus non-survivors, require systematic evaluation.

In this study, we employed a PBI/BM5 mouse model to evaluate serum cytokines as biomarkers for radiation exposure detection, survival outcome prediction, and dose discrimination specifically within the high-dose lethality window (12–16 Gy) of the PBI/BM5 model. Using multiplex cytokine profiling combined with multivariate and classification analyses, we sought to identify cytokine signatures capable of distinguishing radiation exposure status and predicting survival outcome. The primary objective of this study was to characterize the high-radiation-dose circulating cytokine landscape and develop diagnostic signatures capable of distinguishing radiation survival outcomes and quantifying severe exposure levels (12–16 Gy) to guide medical triage. Our findings provide insight into the temporal and dose-dependent behavior of serum cytokines in a realistic radiation exposure model and support their potential utility for radiation biodosimetry and medical triage.

## 2. Results

### 2.1. Survival Curves and Probit Analysis of Male C57BL6 Mice Exposed to Different Doses of PBI/BM5

**Figure 1:**
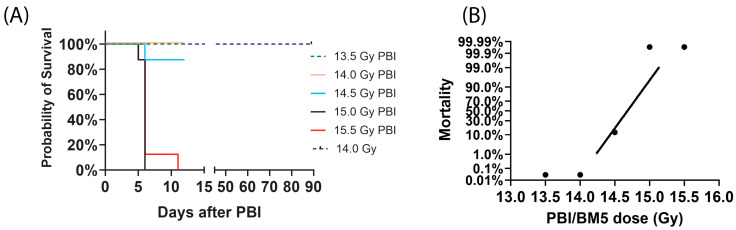
Survival of male C57BL6 mice exposed to different doses of PBI/BM5. (**A**) Kaplan–Meier survival curves demonstrating the dose-dependent lethality of PBI/BM5. For the short-term study (12 days post-irradiation), *n* = 8 mice/group. For the long-term study (90 days post-irradiation), *n* = 20 mice. (**B**) Probit analysis for the short-term study (12-day survival post-irradiation).

We assessed the survivability of male C57BL/6 mice following exposure to varying doses of PBI/BM5 (Figure 1). In the initial study, mice were exposed to doses ranging from 13.5 to 15.5 Gy and monitored for 12 days post-irradiation. A secondary study evaluated long-term survival by monitoring mice for 90 days following a 14.0 Gy dose. As illustrated in Figure 1, doses of 15.0 Gy and 15.5 Gy resulted in 100% lethality, while the 14.5 Gy dose resulted in 12.5% lethality within 12 days. In contrast, doses of 14.0 Gy or lower yielded 100% survival on day 12; furthermore, the separate 14.0 Gy cohort maintained 100% survival through the 90-day observation period. These data indicate that PBI/BM5 doses exceeding 15.0 Gy are uniformly lethal (non-survivors), whereas doses of 14.0 Gy or lower result in total survival for at least 90 days (survivors) (Figure 1A).

To quantitatively define the lethality threshold, a probit regression analysis was performed, yielding an LD_50/12_ of 14.68 Gy (95% confidence interval 14.47–14.97 Gy), confirming a sharp biological transition between 14.0 and 15.0 Gy in survival (Figure 1B).

### 2.2. Principal Component Analysis (PCA) of Serum Cytokines on Each Study Day

To study the serum cytokine changes after high-dose PBI/BM5, male C57BL/6 mice were exposed to 12.0, 13.0, 14.0, 15.0, and 16.0 Gy PBI/BM5 (five radiation doses). On days 1, 3, and 7 after radiation exposure (three time points), separate cohorts of mice were euthanized for terminal blood collection via heart puncture. A baseline control group of 0 Gy (naïve) mice was also included. The experimental design utilized *n* = 4 mice per dose for each time point. Because one mouse in the 16.0 Gy group died prior to the scheduled day 7 collection, a total of 63 mouse serum samples (4 naïve and 59 irradiated) were used for analysis and modeling.

**Figure 2:**
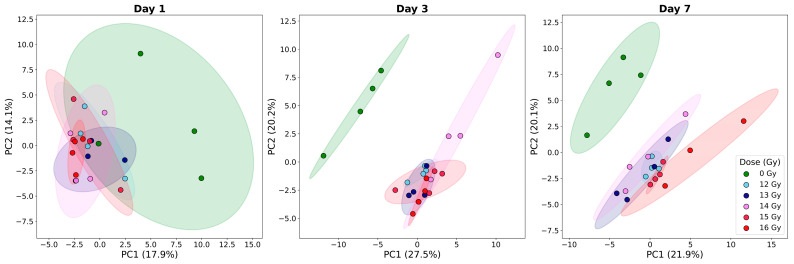
Principal Component Analysis (PCA) of systemic cytokine profiles across radiation doses. PCA plots represent the global cytokine distribution on days 1, 3, and 7 post-irradiation. Each point represents an individual subject, colored by radiation dose: 0 Gy (green), 12 Gy (sky blue), 13 Gy (navy blue), 14 Gy (violet), 15 Gy (crimson-magenta), and 16 Gy (red). Shaded areas represent 95% confidence ellipses for each dose group. PC1 and PC2 percentages indicate the proportion of total variance explained by each component.

To assess the global systemic cytokine response following radiation, Principal Component Analysis (PCA) was performed on log2-transformed cytokine concentrations for each post-irradiation time point (day 1, 3, and 7) relative to the 0 Gy baseline (Figure 2).

The PCA revealed a distinct temporal and dose-dependent evolution of the systemic cytokine profile. On day 1, irradiated groups began to deviate from the 0 Gy control cluster, marking the initiation of the acute systemic response. By day 3, all irradiated groups were clearly separated from the 0 Gy groups, but there was not much separation between the irradiated groups. By day 7, the separation between the 0 Gy and irradiated groups continued, and the separation between the irradiated groups became more obvious. Across all time points, 95% confidence ellipses demonstrated robust clustering within dose groups, particularly at higher radiation levels.

### 2.3. PCA Analysis Comparing 0 Gy (Naïve), Survivors and Non-Survivors on Each Study Day

**Figure 3:**
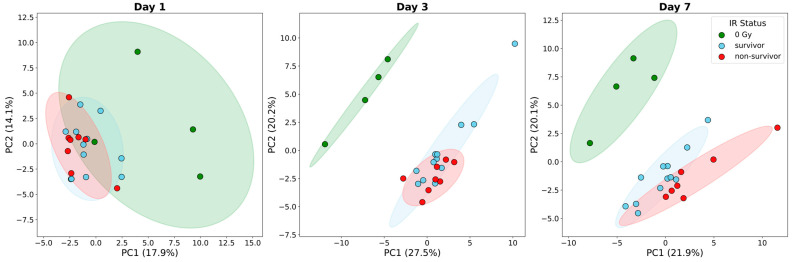
PCA of systemic cytokine profiles by survival status. Three-panel PCA plots illustrate the global distribution of 66 cytokines on day 1, day 3, and day 7. Samples are categorized by survival outcome: 0 Gy (green), survivors (sky blue), and non-survivors (red). Shaded regions represent 95% confidence ellipses.

To evaluate whether global cytokine signatures could distinguish radiation outcomes, we performed PCA comparing 0 Gy (naïve), survivors (12–14 Gy), and non-survivors (15–16 Gy) at each post-irradiation time point (day 1, 3, and 7). The global cytokine profile effectively discriminated between radiation exposure levels and eventual survival outcomes, with the degree of separation evolving over time (Figure 3).

While the initial response on day 1 was similar across all irradiated groups, a distinct outcome-specific signature emerged on day 3, where non-survivors shifted significantly further along PC1. Notably, maximal separation between the survivor and non-survivor clusters was observed on day 7. This widening gap at the later time points likely reflects the terminal systemic divergence of the non-surviving group compared to the stabilizing cytokine profile of survivors.

### 2.4. Heatmap Analysis of Cytokines

**Figure 4:**
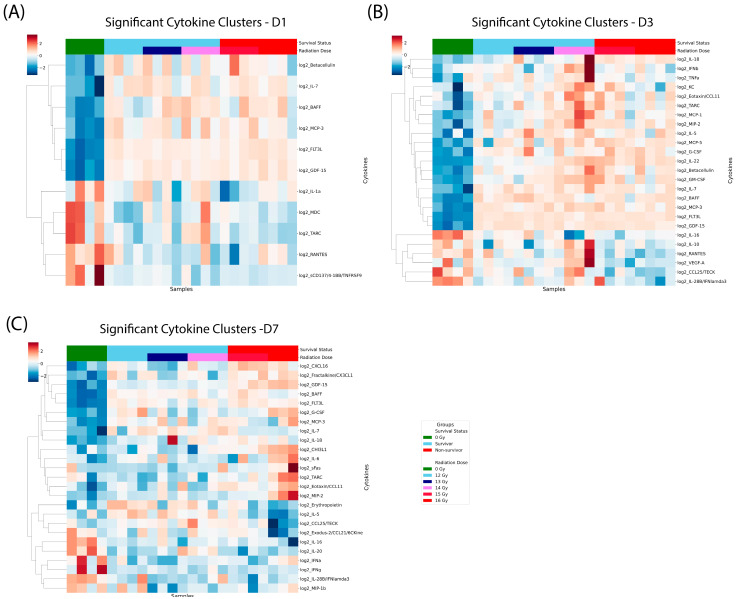
Heatmap of clustered cytokines on each study day based on the survival status and radiation doses. Three heatmap plots illustrate the distribution of clustered cytokines on day 1, 3, and 7 (**A**,**B**,**C**, respectively). Samples are categorized by survival outcome: 0 Gy (green), survivors (sky blue), and non-survivors (red) on the top horizontal band and the radiation doses on the second horizontal band.

To delineate cytokine clusters associated with radiation dose and survival outcomes, hierarchical clustering was performed on significantly altered cytokines at days 1, 3, and 7 post-irradiation (Figure 4). This analysis enabled visualization of global shifts in the peripheral cytokine landscape and identification of distinct molecular signatures linked to radiation dose and survival. Cytokines included in the clustering analysis were those significantly altered at each post-irradiation time point compared to the 0 Gy control group (ANOVA, *p* < 0.05).

On day 1 (Figure 4A), hierarchical clustering revealed a clear segregation between the 0 Gy control group (green) and the irradiated cohorts. The analysis identified 11 significantly altered cytokines. Early after exposure, irradiated mice exhibited broad alterations in cytokines related to hematopoietic stress and immune activation, including FLT3L, GDF-15, MCP-3, and IL-7. While dose-dependent trends were evident, separation between survivors and non-survivors was modest at this early time point, suggesting that day 1 cytokine changes primarily reflect acute radiation injury rather than downstream survival outcomes.

On day 3 (Figure 4B), the systemic response broadened significantly, with 25 cytokines reaching statistical significance. Clustered cytokines were strongly associated with both radiation dose and survival status. Non-survivors clustered together and displayed elevated levels of pro-inflammatory and myeloid-associated cytokines, including G-CSF, MCP-1, MCP-3, KC, IL-6, and IL-1β, whereas survivors showed comparatively lower or more regulated cytokine responses. This divergence indicates an escalating systemic inflammatory response in animals destined for mortality, consistent with progression toward severe hematopoietic and systemic injury.

On day 7 (Figure 4C), cytokine profiles demonstrated the strongest discrimination between survivors and non-survivors. Non-survivors exhibited sustained or further increased expression of inflammatory and chemotactic cytokines (e.g., IL-6, IL-18, MCP-1, KC, G-CSF), while survivors showed partial normalization or attenuation of these responses. Dose-dependent effects were less prominent than survival status at this later time point, suggesting that persistent dysregulated inflammation, rather than radiation dose alone, is closely linked to lethality.

Collectively, these data demonstrate that peripheral cytokine signatures evolve dynamically after irradiation, with day 3 and 7 cytokine clusters strongly associated with survival outcome, supporting their potential utility as prognostic biomarkers of radiation-induced injury severity and lethality.

### 2.5. Development of a Global Cytokine Signature to Predict Radiation Exposure

To identify a robust molecular signature capable of predicting radiation exposure across the acute and sub-acute phases, we applied regularized machine learning techniques to the cytokine profiles of animals on days 1, 3, and 7 post-irradiation. Given the high-dimensional nature of the data (66 assays) relative to the number of samples, we compared the performance of LASSO (L1), Ridge (L2), and Elastic Net regression models and assessed their performance using Leave-One-Out Cross-Validation (LOOCV).

As shown in Table 1, while all three regularization methods achieved perfect classification of radiation exposure (AUC = 1.000), LASSO was selected as the superior approach due to its ability to perform intrinsic feature selection and prevent overfitting. LASSO successfully reduced the 66-cytokine panel into a parsimonious four-marker global signature. Ridge and Elastic Net need a much higher number of cytokines for the signature. Therefore, we picked the LASSO methods for our later analysis.

**Figure 5:**
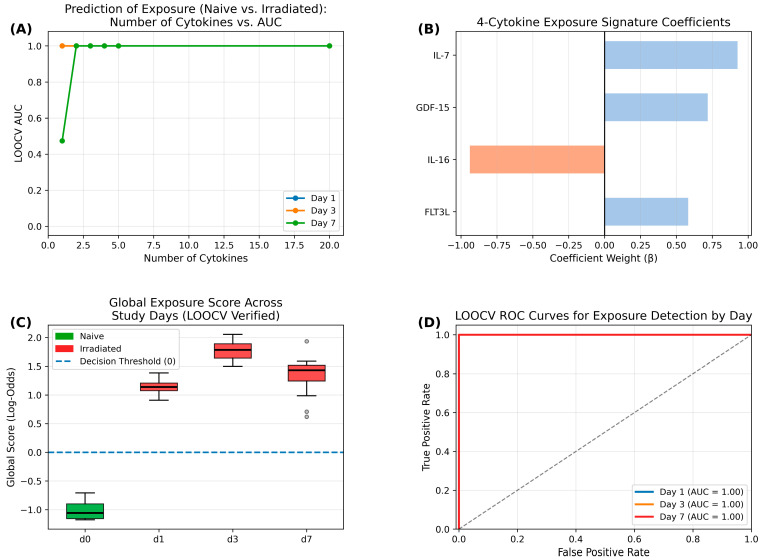
Development and validation of a global 4-cytokine signature to predict radiation exposure. (**A**) Identification of the optimal cytokine panel to predict radiation exposure or not. LOOCV AUC reached 1.0 after 3–4 cytokines and additional cytokines were unnecessary for the model. (**B**) LASSO Coefficients: Relative weights of the four predictive cytokines (GDF-15, FLT3L, IL-16, and IL-7) selected for the global radiation signature. (**C**) S-score Distribution: Composite signature scores across study days (d1, d3, d7) demonstrating clear segregation between naive (green) and irradiated (red) cohorts. (**D**) Diagnostic performance: LOOCV-ROC analysis confirming perfect classification (AUC = 1.000) of irradiated animals across all time points post-exposure.

To develop the global signature to predict radiation exposure (naive vs. irradiated), we utilized a LASSO-based feature selection method integrated with LOOCV using all 63 samples (4 naïve and 59 irradiated mice). A panel of four cytokines—GDF-15, FLT3L, IL-16, and IL-7—was identified as the “global signature” (Figure 5). This specific subset provided perfect predictive accuracy across all study days, with additional cytokines offering only redundant information (Figure 5A). The relative importance of these markers was determined via standardized coefficients, which identified IL-7 and GDF-15 as the most significant positive predictors of radiation status (Figure 5B).

The distribution of S-scores demonstrated a definitive and sustained separation between naive (0 Gy) and irradiated animals throughout the study (Figure 5C). Notably, the signature scores for irradiated animals remained significantly elevated above the naive baseline on all time points. This indicates that the molecular fingerprint captures a conserved biological response to radiation injury that persisted for at least seven days.

The diagnostic performance of this global signature was evaluated using LOOCV-AUC. The four-marker S-score achieved a perfect AUC of 1.000 on days 1, 3, and 7 (Figure 5D), representing absolute discrimination between naive and irradiated cohorts. The robustness of the perfect AUC (1.000) for the exposure model was confirmed via permutation testing (*n* = 1000 iterations), which yielded a null-distribution mean AUC of 0.51 (*p* < 0.001), indicating that the observed classification is not due to random chance (Figure S1). These results demonstrate that a lean, four-cytokine signature provides highly accurate and temporally stable biodosimetry within the male mouse peripheral blood immune environment.

### 2.6. Development of a Global Cytokine Signature to Predict Radiation Survival Status

**Figure 6:**
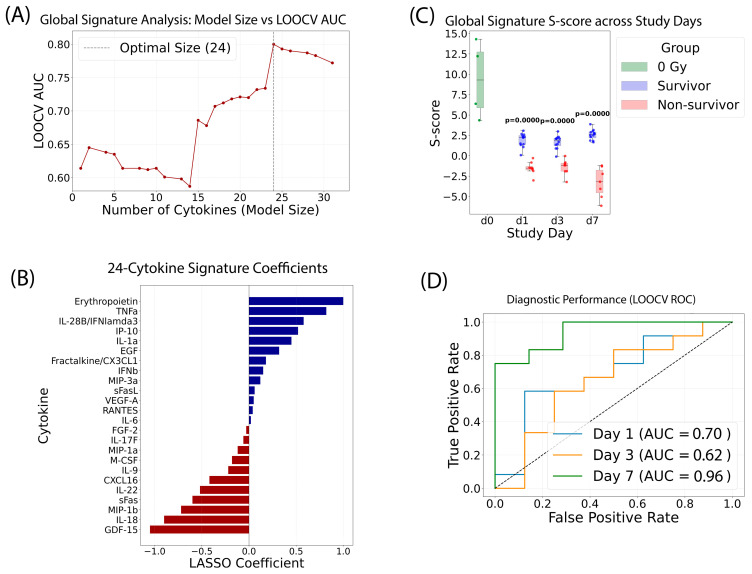
The development and validation of a global 24-cytokine signature to predict radiation survival status. (**A**) Identification of the optimal cytokine panel to predict radiation survivors or non-survivors. LOOCV AUC reached its highest point with 24 cytokines. (**B**) LASSO coefficients: Relative weights of the 24 predictive cytokines selected for the global radiation signature. (**C**) S-score distribution: composite signature scores across study days (d1, d3, d7), demonstrating clear segregation between survivor and non-survivor cohorts. (**D**) Diagnostic performance: LOOCV-ROC analysis confirming classification of survival status across all time points post-exposure.

We employed a similar approach to develop a global cytokine signature for predicting radiation survival status, categorizing animals receiving 12.0–14.0 Gy as survivors and those receiving 15.0–16.0 Gy as non-survivors (utilizing 63 samples, naïve *n* = 4, 12–14 Gy survivors *n* = 36, and 15–16 Gy non-survivors *n* = 23) (Figure 6). The optimal model size was determined by sweeping the LASSO regularization parameter against the LOOCV AUC. A panel of 24 cytokines was identified as the “global signature,” as it achieved the highest predictive accuracy across the entire study period (Figure 6A). Positive predictors associated with survival included Erythropoietin, TNFa, IL-28B, and IP-10, while negative predictors (associated with mortality) included GDF-15, IL-18, and MIP-1b. The relative importance and coefficients for all 24 cytokines (log2 transformed) were detailed in Figure 6B.

The S-score serves as a quantitative metric for this global signature, calculated by summing the standardized log2 concentrations of the 24 cytokines weighted by their respective LASSO coefficients, plus a model intercept (Figure 6C). Mann–Whitney U tests confirmed the signature’s robust ability to distinguish between survival groups on every study day (*p* < 0.001).

The model was validated using the LOOCV method, where each animal’s status is predicted by a model trained independently on all other animals, providing a realistic estimate of performance on unseen data (Figure 6D). The diagnostic performance remained strong and consistent throughout the study: day 1 AUC: 0.70; day 3 AUC: 0.62; and day 7 AUC: 0.96. The exceptionally high AUC on day 7 suggests that as the physiological response to radiation matures, the cytokine signature becomes a nearly perfect predictor of the eventual outcome.

### 2.7. Development of a Global Cytokine Signature to Classify Radiation Doses on Each Study Day

**Figure 7:**
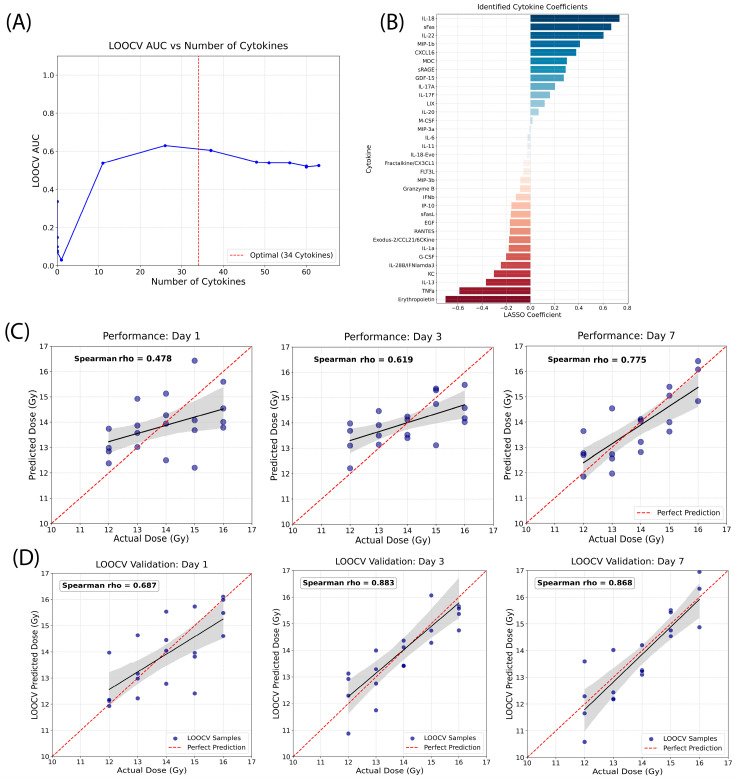
Development and validation of a global 34-cytokine signature to predict radiation doses. (**A**) Identification of the optimal cytokine panel to predict radiation doses. LOOCV AUC reached its highest point with 34 cytokines. (**B**) LASSO coefficients: relative weights of the 34 predictive cytokines selected for the radiation dose estimation. (**C**) Correlation of predicted radiation doses vs. actual radiation doses: composite signature scores across study days (days 1, 3, 7) demonstrating clear linear relationship of radiation doses. (**D**) Quantitative performance: LOOCV-validated Spearman correlation analysis for the reconstruction of radiation doses.

To refine the accuracy of dose estimation within the high-exposure range (12.0–16.0 Gy), we developed a global signature specifically optimized for the irradiated cohorts (*n* = 59 irradiated samples only), excluding naive (0 Gy) controls (Figure 7). By focusing exclusively on the “irradiated-only” space, the model identifies biomarkers that distinguish between levels of severe radiation injury without the dominant signal of the naive-to-irradiated jump.

Using the LOOCV AUC, A panel of 34 cytokines was identified as the “global signature,” as it achieved the highest predictive accuracy across the entire study period (Figure 7A). Positive predictors associated with higher radiation dose included IL-18, sFas, IL-22, and negative predictors included Erythropoietin, TNFa, and IL-13. The relative importance and coefficients for all 34 cytokines are detailed in Figure 7B.

The predicted dose calculated using the developed global signature was compared to the actual radiation doses on each study day. As shown in Figure 7C, Spearman’s rho (measuring the association between two ranked variables) increased from 0.478 on day 1 to 0.619 on day 3, and further to 0.775 by day 7, indicating a progressively stronger monotonic relationship between predicted and actual doses. Consistent with this trend, predicted dose estimates on day 7 clustered more closely around the line of perfect prediction compared with earlier time points. This improved alignment suggests enhanced model performance at later times post-exposure, with more accurate ranking and reduced deviation from actual dose values.

To rigorously validate the robustness of the 34-cytokine signature and prevent overfitting, we performed LOOCV on the entire cohort. The cross-validated results confirmed the high performance of the signature across all time points. The Spearman’s rho increased from 0.687 on day 1 to 0.883 on day 3, and to 0.868 by day 7 (Figure 7D). These high correlation values during cross-validation demonstrated that the 34-cytokine global score was a stable and highly accurate tool for biodosimetry. The Spearman correlation values were higher after LOOCV than before. This may reflect the stability introduced by LASSO regularization together with the use of rank-based correlation. Individual training models may be sensitive to localized variations (e.g., outliers present in only a small number of samples within a specific dose group) rather than capturing a consistent trend across the entire dataset. The LOOCV process may therefore help reduce such noise and produce a more stable signature. Although certain individual cytokines reached a response plateau at these high doses, the 34-cytokine global signature maintained a quantitative resolution by integrating multiple cytokine signals. The Spearman’s rho increased from 0.687 on day 1 to 0.883 on day 3, and to 0.868 by day 7. This suggests that as the sub-acute injury progresses, the integrated molecular signature matures to overcome early saturation effects, providing stable and highly accurate biodosimetry within these dose ranges.

### 2.8. The Concentrations of Significantly Changed Individual Cytokines

**Figure 8:**
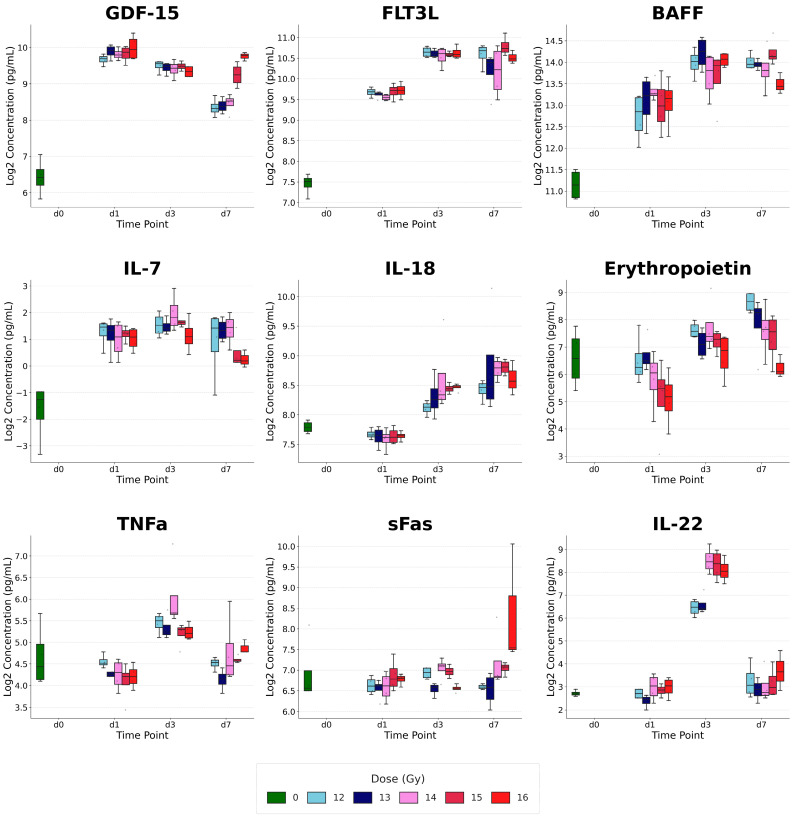
Individual cytokine changes following PBI exposure. Relative to the 0 Gy control group (green), irradiated groups (12–16 Gy) exhibit progressive divergence. Color-coded groupings (sky blue to red) illustrate the dose-escalation effect within each time point (d1, d3, and d7).

To evaluate individual cytokine responses to PBI, two-way ANOVA identified a subset of cytokines—GDF-15, FLT3L, BAFF, IL-7, IL-18, Erythropoietin, TNFα, sFas, and IL-22—that exhibited distinct temporal and dose-dependent profiles, reflecting the complex, dynamic inflammatory environment induced by high-dose radiation (Figure 8).

#### 2.8.1. Cytokines with Significant Dose × Time Interactions

Several cytokines displayed strong interactions between dose and time, indicating that the magnitude of the radiation effect depended on the post-exposure interval. GDF-15 showed the most robust response, with highly significant main effects for both time and dose and a strong interaction (*p* < 0.0001). IL-22 and sFas also exhibited significant interaction effects (*p* = 0.0028 and *p* = 0.0018, respectively). IL-22 remained relatively stable at Day 1 but showed dramatic, dose-stratified increases by Day 3. In contrast, sFas—a marker of extrinsic apoptosis—showed unique temporal patterns across dose groups, with elevations evolving differently over the seven-day period. These dynamic profiles highlight cytokines that are both sensitive to dose and reflective of evolving physiological responses over time.

#### 2.8.2. Time-Dominant and Dose-Dependent Responders

Several cytokines primarily exhibited pronounced temporal changes, often accompanied by overall dose-dependent elevations. Hematopoietic and stress markers—FLT3L, BAFF, and Erythropoietin—were significantly influenced by time (*p* < 0.0001). FLT3L and BAFF increased progressively from day 1 to day 7, likely reflecting ongoing depletion of hematopoietic progenitor cells and B-cell compartments. Erythropoietin demonstrated significant main effects of both time and dose (*p* = 0.0012), indicating an early and sustained erythropoietic stress response following PBI.

Pro-inflammatory signaling cytokines, including IL-18 and TNFα, showed strong time-dependent increases (*p* < 0.0001). TNFα also exhibited a modest dose effect (*p* = 0.0488), peaking at Day 3 across most irradiated groups. IL-18 continued to rise through Day 7, suggesting persistent inflammasome activation in the sub-acute phase. IL-7, associated with lymphoid homeostasis, showed a significant main effect of time (*p* = 0.0059), with concentrations fluctuating over the observation window, potentially reflecting the body’s attempt to regulate lymphoid recovery following radiation-induced lymphopenia.

Collectively, these results demonstrate that cytokine responses following PBI are highly dynamic and cytokine-specific, with distinct temporal and dose-dependent patterns. Cytokines with significant dose × time interactions, such as GDF-15, IL-22, and sFas, highlight sensitive markers of radiation exposure that evolve differently across doses and post-exposure intervals. Time-dominant and dose-dependent responders, including FLT3L, BAFF, Erythropoietin, IL-18, TNFα, and IL-7, reflect ongoing physiological processes such as hematopoietic stress, inflammatory signaling, and lymphoid regulation. Together, these patterns provide a high-resolution view of the animal’s physiological state after high-dose radiation, illustrating both acute and sub-acute responses and offering potential biomarkers for radiation exposure and tissue-specific effects.

The inter-individual biological variability was assessed by calculating the Coefficient of Variation (CV) for the nine significantly changed cytokines (Table S1). The average CV was 10.84%, with 88% of the key markers (8/9) exhibiting a CV < 15%. IL-7 showed a higher CV (49.5%), which was determined to be a mathematical artifact caused by mean log2 values approaching the lower limit of detection, rather than excessive biological noise. Overall, these low dispersion measures support the high reproducibility of the cytokine signatures across the high-dose range

## 3. Discussion

The results of this study establish a multi-tiered molecular diagnostic framework leveraging specialized cytokine signatures to address three critical challenges in radiation emergency medicine: rapid exposure screening, high-resolution dose estimation, and accurate survival prognosis. By optimizing three distinct panels—a 4-cytokine signature for exposure, a 34-cytokine signature for dose, and a 24-cytokine signature for survival—we demonstrate that the systemic inflammatory response contains sufficient information to guide complex triage decisions from the hyper-acute phase through the first week post-exposure. Our data suggest that serum cytokines measured in the early days after irradiation may serve as valuable prognostic indicators for nonlethal PBI/5BM exposures during the subacute phase, as illustrated in Figure 1 (14.0 Gy, monitored for up to 90 days).

### 3.1. The Four-Cytokine “Binary Screen” for Rapid Triage

In mass-casualty scenarios, the immediate priority is segregating the “concerned citizens not needing care” from individuals requiring urgent intervention [3]. The sparse four-cytokine panel effectively classifies exposure (naïve vs. irradiated), reflecting a massive and distinct shift in systemic physiology following radiation injury. Among the four cytokines, GDF-15 and FLT3L are well known to be highly elevated in the blood after radiation exposure [13,17,18], whereas the changes in circulating IL-7 and IL-16 have not been reported. Their dramatic elevation/decrease following PBI highlights the all-or-nothing nature of the molecular response. The simplicity and efficiency of this model make it highly amenable to rapid, point-of-care lateral flow assays, enabling immediate field triage.

### 3.2. The 24-Cytokine Prognosticator: Assessing Biological Effective Dose

Physical dose alone does not always predict survival, particularly in partial-body irradiation scenarios where individual radiosensitivity varies. The 24-cytokine survival signature evaluates the “Biological Effective Dose,” identifying patients whose inflammatory response indicates a high risk of mortality. This panel incorporates acute-phase cytokines (e.g., IL-6, TNFα) and hematopoietic regulators (FLT3L, IL-7) to detect tipping points where intervention is critical. By distinguishing individuals with identical physical doses but divergent survival probabilities, this signature enables prioritization of limited medical resources, such as cytokine therapy or bone marrow transplantation.

Radiation dose and survivability were tightly linked in this model (e.g., below 14 Gy are survivors, whereas ≥15 Gy are non-survivors). However, the model was trained using cytokine features rather than radiation dose as an input variable. Therefore, the classifier learns patterns in the cytokine response associated with survival outcomes rather than a predefined dose threshold. In addition, biological responses to radiation are not perfectly uniform within a given dose group; cytokine levels vary among individuals due to differences in inflammatory and stress responses. The model leverages these biological variations to identify signatures associated with survival versus non-survival. Thus, although survival correlates with dose at the population level, the predictive features used by the model reflect host biological responses to radiation injury rather than the dose boundary itself.

### 3.3. The 34-Cytokine Signature as a Precision Biodosimeter

Once exposure is confirmed, accurate dose estimation becomes essential for guiding clinical management. The 34-cytokine panel allows discrimination between 12.0 Gy and 16.0 Gy. Key markers, such as IL-22 (reflecting GI barrier integrity [19]) and Erythropoietin (reflecting hematopoietic stress and hypoxia [20,21]), contribute to this nuanced resolution. Importantly, the predictive accuracy of this model improves over time, with Spearman’s ρ increasing from Day 1 to 0.868 on Day 7 (LOOCV), suggesting that the molecular “memory” of radiation exposure strengthens as tissue damage progresses.

The ability to discriminate between 12.0 and 16.0 Gy, despite the saturation of individual markers like GDF-15, was attributed to the inclusion of “secondary” responders such as IL-22 and Erythropoietin. These markers provide a more nuanced resolution of organ-specific damage (e.g., GI and hematopoietic stress) that continues to vary at extreme dose levels.

### 3.4. Biological Convergence and Temporal Evolution

Cytokines such as GDF-15, FLT3L, and IL-18 appear across multiple models, representing a universal “radiation signature” encompassing DNA damage, hematopoietic depletion, and inflammasome activation. Conversely, markers unique to specific models, like IL-22 in the survival panel or Erythropoietin in the dose panel, reflect specialized physiological processes, including gut repair and erythropoietic stress. The significant dose × time interactions observed for sFas and IL-22 underscore the importance of temporal analysis, confirming that a single biomarker threshold is insufficient for robust biodosimetry. While some cytokines in our signatures are part of a generic systemic inflammatory response (such as IL-6), the integration of biomarkers like FLT3L, IL-18, and IL-22 provides specificity for radiation-induced hematopoietic and gastrointestinal injury. The dose × time interactions identified in our individual cytokine analysis (e.g., for sFas and IL-22) suggest that these molecular signatures represent a coordinated response to the specific timing and magnitude of radiation injury, rather than a non-specific inflammatory signal.

The identified cytokines may represent complementary biological pathways of radiation injury rather than redundant signals. For example, FLT3L and IL-7 reflect hematopoietic and immune system depletion [22,23], and GDF-15 indicates DNA damage and overall genotoxic stress [24]. IL-16 in the radiation response has been scarcely studied, but similar to our data, it has been shown that IL-16 levels decreased after radiotherapy in patients [25]. By integrating these mechanistically independent signals, the LASSO-derived signatures achieve greater stability and predictive power, effectively “de-noising” individual biological variability to provide a robust assessment of radiation exposure and its subsequent clinical trajectory.

### 3.5. Bridging the Gap: Serum Cytokine Responses Across Low, Intermediate, and Lethal Radiation Doses

It will be a great advantage if the cytokines behave the same way across the low, intermediate and lethal radiation doses. Several biomarkers identified in our study behave in this way. For example, FLT3L and IL-18 have been characterized extensively as robust radiation biomarkers across multiple species and dose ranges. Plasma FLT3L levels demonstrated a radiation dose-dependent increase during the first week after 2–8 Gy TBI in nonhuman primates, and their level peaked at 2–3 weeks post-TBI, depending on the radiation doses [22]. In mice exposed to 4, 7.5 and 11 Gy, with either 25, 50, 75, or 100% of bone marrow being irradiated, the peak plasma FLT3L was determined by the percentage of irradiated bone marrow and the radiation doses [26]. It has been shown that plasma FLT3L levels at 24, 48 and 72 h consistently predicted radiation doses (1–8 Gy) received in mice, and the FLT3L concentrations are similar to our data (from ~200 pg/mL at baseline to ~2000 pg/mL at the highest levels) [27]. Plasma FLT3L concentrations were shown to directly reflect the radiation-induced marrow damage during fractionated local radiotherapy [28]. Furthermore, plasma FLT3L has been used clinically in a real radiological accident victim to evaluate radiation damage [29]. Our lab’s previous work has shown that circulating IL-18 is a biomarker of TBI in various animal models such as mice, minipig and nonhuman primates; particularly, mouse serum IL-18 levels were significantly elevated in the dose ranges of 5–12 Gy, and peaked on d3 within a two-week period post-radiation [15], and urine IL-18 levels also peaked on d3 for 6.5 and 8.5 Gy TBI in nonhuman primates [30]. Studies have demonstrated that GDF-15 expression increases in human fibroblasts at doses from 100 mGy up to 2 Gy, suggesting its potential utility as an early biomarker across a wide dose range [24]. Another cytokine, IL-7, has been shown to have a continuous increase from day 5 to day 21 after 8 Gy X-ray irradiation in rat thymus [31].

On the other hand, the informative cytokines reflecting low-dose and high-dose radiation may be different. For example, it has been shown that there are differential gene expression patterns after acute exposure to low and high doses of X-ray (0.1, 2.0 and 10.0 Gy) in a reconstituted human skin tissue [32].

The fact that cytokines such as FLT3L, IL-18 and GDF reflect exposure at low to intermediate radiation doses (<12 Gy) strongly complements our current high-dose data (12–16 Gy), supporting the use of these biomarkers across a complete diagnostic range (0–16 Gy). Our study extends this knowledge by demonstrating their efficacy within a clinically relevant PBI/BM5 model and by identifying novel markers, such as IL-7 and IL-16, which further enhance the predictive power for high-dose lethality and dose reconstruction. Future study, including low-dose samples, will help to prove whether the established panels in the current study hold true across radiation doses.

### 3.6. Limitations

Several limitations should be noted. First, this study was deliberately focused on a high-dose window (12–16 Gy) bracketing the lethality threshold of the PBI/BM5 model. While this scope is scientifically justified and transparently stated throughout the manuscript, it means the present data do not provide information on cytokine responses at lower or moderate radiation doses and do not allow identification of the dose at which cytokine levels begin to deviate from baseline. Future studies are required to validate these panels across low-to-intermediate dose ranges, as well as chronic or mixed-field exposure scenarios. Second, the cytokine response plateau observed between 12 and 16 Gy indicates some saturation of inflammatory pathways at the highest doses, which may limit discrimination among very high doses. The use of a high-dimensional 34-cytokine panel partially mitigates this by capturing collective, non-saturated variance across the systemic immune environment. Third, the survival transition between 14.0 and 15.0 Gy is a model-specific feature of the PBI/BM5 design, in which 5% bone marrow sparing enables animal survival below a critical dose threshold but not above it. This does not imply an abrupt transition in cytokine biology; as demonstrated in the PCA and heatmap analyses, cytokine responses changed in a graded, dose-dependent manner across the 12–16 Gy range. The LD_50_/_12_ derived by probit analysis is a standard parametric estimate of the dose–mortality midpoint and should not be interpreted as evidence for a mechanistic cytokine threshold. Fourth, biological variability between animals may influence both cytokine measurements and survival outcomes. Inter-individual variability was minimized through the use of an inbred murine strain, but variations in individual mouse body size and positioning during irradiation may represent an additional source of dosimetric uncertainty that was not fully characterized. While phantom-based dosimetry confirmed field uniformity, per-animal dosimetric uncertainty should be considered when interpreting inter-individual cytokine variability. Fifth, while the 4-cytokine panel is well-suited for field deployment, the 34-cytokine panel currently requires laboratory infrastructure, complicating point-of-care implementation. Sixth, this study did not include an independent validation cohort. While LOOCV provided a rigorous internal assessment of model stability, future studies should validate the established 4-, 24-, and 34-cytokine signatures in separate cohorts and diverse populations to ensure broader applicability. Finally, although murine models provide important mechanistic insights, species-specific differences in radiation sensitivity and immune responses limit direct extrapolation of these findings to human radiation exposure scenarios. This study is further limited to male mice only; factors such as sex, age, and pre-existing comorbidities in human populations will introduce significantly greater variability. Nonetheless, several cytokines identified here—including FLT3L, GDF-15, G-CSF, IL-6, and IL-18—have been reported in human and nonhuman primate radiation exposure data, supporting the biological plausibility of future translational work. Validation in nonhuman primate models and human biobank samples from radiation-exposed cohorts will be essential before clinical deployment.

## 4. Materials and Methods

### 4.1. Ethics Statement

The animals were housed in an Association for Assessment and Accreditation of Laboratory Animal Care (AAALAC)-approved facility at the Uniformed Services University of the Health Sciences (USUHS). All animal study procedures, including housing, irradiation, survival study, and blood/tissue collection, were reviewed and approved by the USUHS Institutional Animal Care and Use Committee, and all experiments were performed in accordance with the guidelines and regulations from the USUHS-IACUC and the USUHS Department of Laboratory Animal Resources (DLAR).

### 4.2. Mice and Animal Care

C57BL6 mice (male), at 12–14 weeks of age at the time of irradiation, were sourced from the Jackson Laboratory (Bar Harbor, ME, USA). Animals were randomly assigned to all experimental groups and housed in an AAALAC-approved facility at USUHS. The animal rooms were maintained at 20–26 °C with 30–70% humidity on a 12 h light/dark cycle. Commercial rodent chow (Harlan Teklad Rodent Diet 8604, Inotiv, Indianapolis, IN, USA) was available ad libitum, as was acidified water (pH = 2.5–3.0) to control opportunistic infections. There were no statistical body weight differences between the study groups prior to radiation exposure.

### 4.3. Irradiation

PBI was carried out as previously described [33] at an estimated dose rate of 2.8 Gy/min using an Elekta Infinity^TM^ linear accelerator (LINAC) (4 MV X-ray) (Elekta, Stockholm, Sweden). Detailed dosimetry was carried out to determine uniformity of the field, inline penumbra, and the radiation dose distribution to the targeted area, as well as the excluded part of the body, with in-run monitoring of the delivered dose. Ionization chambers, alanine dosimeters with traceability to national standards, and mouse phantoms were used to measure radiation doses. Prior to irradiation of mice, the beam output was confirmed through ion chamber measurements (30,013, PTW) calibrated at a National Institute of Standards and Technology (NIST)-traceable, accredited dosimetry calibration laboratory, at the field center. The ion chamber dosimetry was based on the calculation of absorbed dose rate that was described in the American Association of Physicists in Medicine (AAPM) Task Group (TG)-51 protocol [34]. The mice were anesthetized during irradiation using ketamine + xylazine injection. For PBI/BM5, mice were exposed to specified doses of radiation to the whole body except the hind limbs’ fibulae, tibiae, and feet (which constituted about 5% of bone marrow [35,36]). All animals were transferred back to their respective cages for recovery from anesthesia. The mice were provided with free access to food and acidified water and monitored daily (up to three times a day during the critical period d3–d10) for body weight loss, ruffled fur, or any behavioral abnormalities. Mice showing signs of moribundity (significant weight loss, difficulty in breathing and moving, inability to stand, etc.) according to predetermined parameters [37] were euthanized immediately via CO_2_ inhalation exposure and cervical dislocation according to the institutional IACUC guidelines.

The dose mapping for the irradiation geometry used in this study was based on alanine electron paramagnetic resonance. The alanine dosimeters were calibrated at the National Physical Laboratory (Teddington, UK), in terms of absorbed dose-to-water, with an uncertainty of +/−2.2%. All irradiations employed static beams with the same table (height: 10.4 cm, rotation: 90 degrees) and collimator (inline direction jaws: 7 cm, crossline direction multileaf collimator: 20 cm) positions. The positional accuracy of the Elekta Precise table height was within 1 mm, table rotation was within 0.5 degrees, and Agility collimator was within 1 mm. The uncertainty in the table height, table rotation, inline collimator position, and crossline collimator position were 0.96%, 0.56%, 1.4%, and 0.5%, respectively. The effect of variations in individual mouse positions on dosimetry was not investigated.

During the dose mapping measurements, irradiation geometry was replicated as closely as possible to that used for the experimental irradiation of mice. The mouse phantoms were rectangular Plexiglas blocks (6 cm L × 3 cm W × 1.5 cm H). Three alanine pellets were inserted into each mouse phantom at positions corresponding to the head, abdomen, and pelvis. The table position, radiation field size, and distance from the source used during mapping were the same as those used for the irradiation of mice. The number of monitor units required to deliver the target dose was based on the average dose rate to the head and abdomen pellets. Variations in the shape and size of individual mice that may affect the absorbed dose were not considered in the current study.

### 4.4. Serum Cytokine Assay

Whole blood was collected via heart puncture at a single terminal time point (day 1, 3, or 7) for each subject; no serial blood sampling was performed, ensuring that each data point in the statistical analysis represented an independent biological subject. Serum was prepared in BD Microtainer Gold tubes as described before [38,39]. The prepared serum was analyzed to determine the concentrations of 68 cytokines using the Mouse Cytokine/Chemokine 68-Plex Discovery Assay^®^ Array (MD68) (Eve Technologies, Calgary, AB, Canada). These targets include BAFF, Betacellulin, CCL25/TECK, CHI3L1, CXCL16, EGF, Eotaxin/CCL11, Erythropoietin, Exodus-2/CCL21/6Ckine, FGF-2, FLT3L, Fractalkine/CX3CL1, G-CSF, GDF-15/MIC-1, GM-CSF, Granzyme B, IFNα, IFNβ, IFNγ, IL-1α, IL-1β, IL-2, IL-3, IL-4, IL-5, IL-6, IL-7, IL-9, IL-10, IL-11, IL-12p40, IL-12p70, IL-13, IL-15, IL-16, IL-17A, IL-17F, IL-18, IL–20, IL-22, IL-28B/IFNλ3, IL-31, IL-33/NF-HEV (mature), IP-10/CXCL10, KC/GROα/CXCL1, LIF, LIX/CXCL5, MCP-1/CCL2, MCP-2/CCL8, MCP-3/CCL7, MCP-5/CCL12, M-CSF, MDC/CCL22, MIG/CXCL9, MIP-1α/CCL3, MIP-1β/CCL4, MIP-2/CXCL2, MIP-3α/CCL20, MIP-3β/CCL19, RANTES/CCL5, sCD137/4-1BB/TNFRSF9, sFas/TNFRSF6, sFasL, sICAM-1, sRAGE, TARC/CCL17, TNFα, and VEGF-A.

Separately, serum IL-18 and IL-18BP were quantified using mouse IL-18 (Medical Biological Laboratories, Tokyo, Japan) and mouse IL-18BP (Cloud-Clone Corp, Houston, TX) ELISA kits, as previously reported [39].

The IL-18 results measured using the Eve assay were labeled IL-18-Eve, while the IL-18 results measured using the Medical & Biological Laboratories ELISA assay were labeled IL-18.

### 4.5. Serum Cytokine Analysis and Prediction Signature Development

Serum cytokine data pretreatment: Among the 68-plex Eve cytokine panel, IL-3, IL-4, and LIF were below the detection limit, while all MCP-2 exceeded the upper detection range. These four cytokines were therefore excluded from downstream analyses. For cytokines with values below the detection limit, concentrations were imputed as the lower limit of detection divided by sqrt(2). Therefore, a total of 66 cytokines (64 from the multiplex + IL-18 and IL-18BP) were used for data analyses performed using Python (v3.10).

#### 4.5.1. PCA Analysis

PCA was performed on log2-transformed cytokine data at days 1, 3, and 7 post-irradiation. Data were mean-centered and scaled to unit variance. The first two principal components (PC1 and PC2) were extracted to visualize the sample distribution. Samples were color-coded by survival status (0 Gy: green; survivor: sky blue; non-survivor: red) and radiation dose (0–16 Gy: green to red gradient).

#### 4.5.2. Hierarchical Clustering and Heatmaps

One-way ANOVA (*p* < 0.05) was used to identify cytokines significantly altered across experimental groups at each time point. Significant markers were standardized via Z-score normalization. Unsupervised hierarchical clustering was executed using Ward’s linkage and Euclidean distance. Samples were organized by survival status and doses to facilitate visual comparison using Seaborn (v.0.13.2; https://seaborn.pydata.org) and SciPy (v.1.17.0; https://scipy.org) [accessed on 1 February 2026].

#### 4.5.3. Global Signature Development and LASSO Regression

To establish a molecular framework for radiation biodosimetry, we pooled cytokine data from all post-irradiation time points (days 1, 3, and 7) to create a “time-independent” global signature. This approach addressed the *p* > n dimensionality challenge and reduced overfitting to temporal fluctuations. Using Scikit-learn in Python, we evaluated Ridge, LASSO, and Elastic Net algorithms to compare their predictive performance and feature selection capabilities. Model performance and resistance to inter-individual variability were rigorously judged using Leave-One-Out Cross-Validation (LOOCV), which iteratively tested the models on unseen samples. LASSO (Least Absolute Shrinkage and Selection Operator) was selected as the final architecture due to its ability to generate parsimonious signatures by shrinking non-contributing coefficients to zero. To prevent over-optimistic performance estimates and data leakage, the LASSO regularization parameter (λ) was tuned independently within each iteration of the LOOCV process. The optimal was selected based on the minimum cross-validated error of the training subset, ensuring that the model’s feature selection and shrinkage were blinded to the held-out test subject. This process resulted in three distinct diagnostic models:

#### 4.5.4. Binary Exposure Detection (Four-Marker Signature)

To distinguish irradiated subjects from controls regardless of dose. This parsimonious model was optimized for diagnostic speed and stability, with performance validated via Area Under the Receiver Operating Characteristic (AUC-ROC) curves.

#### 4.5.5. Survival Prediction (24-Marker Signature)

To predict animal mortality (survivor vs. non-survivor), we utilized a logistic regression workflow on Z-score-normalized log2 data. This identified an optimal 24-cytokine panel, from which a linear predictor (S-score) was derived: S = Constant + ∑Wi⋅log2(Ci).

S > 0 predicts survival, while S < 0 predicts non-survival. The model achieved significant separation (*p* < 0.0001, Mann–Whitney U) and an AUC of up to 0.96.

#### 4.5.6. Dose Reconstruction (34-Marker Signature)

For quantitative dose assessment, iterative feature selection identified a 34-cytokine subset as the optimal configuration. Model accuracy was verified using Spearman’s rank correlation (ρ) to assess the relationship between model-predicted values and actual administered doses (12–16 Gy).

#### 4.5.7. Individual Cytokine Change Analysis

To assess changes across the 16 experimental groups, a two-way ANOVA was employed, treating radiation dose and time post-exposure as independent factors. Interaction effects (dose × time) were analyzed to identify dynamic response profiles. Post hoc analyses were conducted using Dunnett’s test (vs. 0 Gy control) or Tukey’s HSD (pairwise comparisons). All visualizations were generated using Matplotlib (v3.10.8; https://matplotlib.org) [accessed on 1 February 2026] and Seaborn.

## 5. Conclusions

By leveraging a machine learning-driven approach, we successfully transitioned from a complex 66-plex panel to highly focused, parsimonious biosignatures capable of addressing three critical diagnostic needs: exposure detection, survival prediction, and dose reconstruction. This study demonstrates that peripheral blood cytokine profiles serve as robust, time-independent tools for high-dose radiation triage, survival prediction, and dose discrimination within the near-lethal exposure window (12–16 Gy) of the PBI/BM5 model. These findings are explicitly bounded to this high-dose range; validation across a broader dose spectrum, in additional species, and in independent cohorts is required before clinical deployment.

## Figures and Tables

**Table 1:** Comparison of three machine learning techniques to predict radiation exposure.

Method	Mean AUC	Number of Features in Signature	Key Advantages
LASSO (L1)	1.000	4	Highest sparsity; eliminates redundant features
Ridge (L2)	0.997	66	Minimal feature reduction; keeps all noise
Elastic Net	1.000	25+	Balanced approach, but more complex than LASSO.

## Data Availability

The data presented in this study are available from the corresponding authors upon request.

## Supplementary Materials

The following supporting information can be downloaded at: https://www.mdpi.com/article/10.3390/ijms27073213/s1.

## Abbreviations

The following abbreviations are used in this manuscript:

ARS	Acute Radiation Syndrome
PBI/BM5	Partial-body irradiation with 5% bone marrow sparing
Gy	Gray (unit of absorbed radiation dose)
PCA	Principal Component Analysis
LASSO	Least Absolute Shrinkage and Selection Operator
LOOCV	Leave-One-Out Cross-Validation
AUC-ROC	Area Under the Receiver Operating Characteristic Curve
ANOVA	Analysis of Variance
FLT3L	FMS-like tyrosine kinase 3 ligand
GDF-15	Growth Differentiation Factor 15
IL	Interleukin
sFas	Soluble Fas
TNFα	Tumor Necrosis Factor-alpha
BAFF	B-cell Activating Factor
