# Supplementary figures and images for: Cytokine Profiling for the Prediction of Lethality and High-Dose Exposure in a Murine Partial Body Irradiation Model
Source: Int J Mol Sci. 2026 Apr 1;27(7):3213. doi: 10.3390/ijms27073213 (PMC13073184; doi:10.3390/ijms27073213)

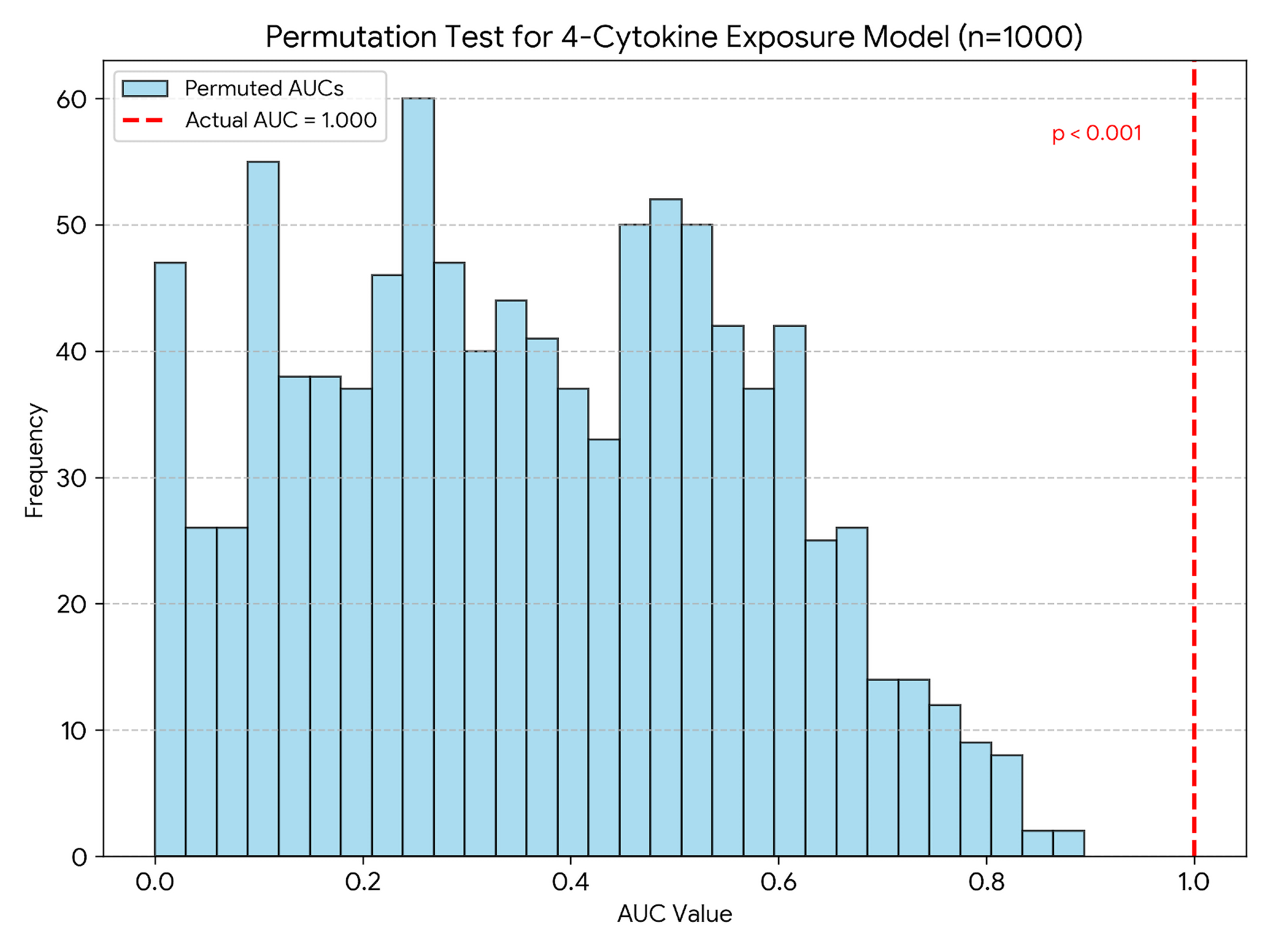

Supplement: Supplementary file 1 [file ijms-27-03213-s001.zip › Figure S1 permutation.tif]
